# Editorial: Integrated Multi-modal and Sensorimotor Coordination for Enhanced Human-Robot Interaction

**DOI:** 10.3389/fnbot.2021.673659

**Published:** 2021-04-15

**Authors:** Bin Fang, Cheng Fang, Li Wen, Poramate Manoonpong

**Affiliations:** ^1^Department of Computer Science and Technology, Beijing National Research Center for Information Science and Technology, Tsinghua University, Beijing, China; ^2^University of Southern Denmark Robotics, The Maersk Mc-Kinney Moller Institute, University of Southern Denmark, Odense, Denmark; ^3^School of Mechanical Engineering and Automation, Beihang University, Beijing, China; ^4^Embodied Artificial Intelligence and Neurorobotics Lab, University of Southern Denmark Biorobotics, The Maersk Mc-Kinney Moller Institute, University of Southern Denmark, Odense, Denmark; ^5^Bio-Inspired Robotics and Neural Engineering Lab, School of Information Science and Technology, Vidyasirimedhi Institute of Science and Technology, Rayong, Thailand

**Keywords:** sensorimotor coordination mechanism, multimodality, human-robot interaction, bio-inspired models, coadaptation in interaction

With the widespread application of intelligent robots and all kinds of sensors in our lives, human-robot interaction has become a significant research direction. Traditional human-robot interaction is often based on a single modality through a few sensors. However, with the increasing complexity of application scenarios, it is difficult to improve the interaction performance based on a single modality. Therefore, it is necessary to study human-robot interaction based on multi-modal approaches.

Integration of the sensory and motor systems allows human beings to make full use of sensory information to take meaningful motor actions. Multi-modality is often needed to gather various types of sensory information to better understand the context of complicated tasks in an ever-changing environment; for example, a cognitive or physical human-robot interaction scenario. Integrated multi-modal and sensorimotor coordination are therefore crucial for humans in tackling complex tasks requiring interactions or collaborations between humans and robots, as can be increasingly observed in many different domains, such as industry, healthcare, and rehabilitation.

The new generation of robots are meant to gradually participate in our lives and coexist in human living environments. This has encouraged investigation into technologies enabling effective interactions between humans and robots. The goal of human-robot interaction research is to make robots capable of operating in human-centered spaces to enhance work efficiency, introduce flexibility and adaptability in solutions, and improve the quality of human life.

Investigating the underlying mechanisms of multi-modal sensorimotor integration and coordination in humans will provide insight into the adaptability and compliance of human intelligence with biomechanical sensorimotor control. It will also advance the development of an equivalent robot partner through the transfer and deployment of knowledge learned from humans toward enhanced human-robot interaction. In this challenging scenario, both human and robot partners are expected to perceive their own state and that of their partner during interaction in a multi-modal manner. This involves observing the posture of the partner, monitoring the forces transmitted through a commonly operated object, communicating verbally to understand the intention of the partner, adjusting the collaboration to accomplish the task in an ergonomic and efficient way, and assigning the task roles according to the cognitive and physical strengths of the human and robot partners.

This special issue contains 16 research articles that fourteen research articles focusing on human-robot tasks, one on the object recognition and one on the systematic review.

He and Mathieu studied the extraction of different signals from a single muscle, with the potential to control the origin of multiple degrees of freedom in modern upper limb electromyography (EMG) prosthesis, demonstrating that the characteristics of biceps muscle synergy may promote the control of upper limb EMG prosthesis. In order to improve the quality of life in patients with severe dyskinesia, a brain-computer interface (BCI) for manipulator control was studied by Zhu et al., who proposed an asynchronous hybrid BCI with the ability to complete complex manipulator control tasks.

Wen et al. proposed a feature classification method based on visual sensors in dynamic environments. For detecting objects, a double projection error algorithm is proposed that combines texture and region constraints to achieve accurate feature classification in four different environments. The algorithm can classify static and dynamic feature objects and optimize the conversion relationship between frames only through visual sensors.

With the aging population and consequent increase in hemiplegic patients due to accidents, the provision of rehabilitation training has become a meaningful topic. Bong et al. developed a novel robotic system with a muscle-to-muscle interface to enhance the rehabilitation of post-stroke patients. The system can customize and adjust the rehabilitation training according to the different stages of motor recovery in stroke patients and can run in three different modes, allowing passive and active exercise for effective rehabilitation training. Zuo et al. proposed a structure of wearable parallel mechanisms with sufficient motion isotropy, high force transfer performance, and large maximum torque performance to cover all possible motion ranges in the human ankle joint complex, making it suitable for ankle joint rehabilitation. In terms of rehabilitation training equipment, the exoskeleton has been studied by many researchers. Peng et al. proposed a new data-driven optimal control strategy for adapting to the unpredictable disturbance of different hemiplegic patients. Fang et al. proposed a temporal convolutional network based gait recognition and prediction model to recognize and predict the actions of the exoskeleton wearer. Shi et al. first linearized and discretized the constraint conditions of model predictive control by using a third-order Taylor-type numerical differential formula, extending it to a lower limb rehabilitation robot to realize human-computer interaction control and intention recognition in active rehabilitation training.

Jin et al. proposed a container target recognition framework based on acoustic signals in an open environment using the kernel k-nearest neighbor algorithm. The dynamic contact method was used to collect the acoustic signal in the container to solve the problem of object recognition (Jin et al.).

Jiao et al. combined the attitude estimation system with an intelligent control structure to make the unmanned aerial vehicle (UAV) perform the detection task stably, and proposed an intuitive end-to-end interaction system, which could control the UAV according to the natural posture of the human body.

In order to make robot learning surpass human demonstration and task completion under unknown conditions, Cao et al. proposed the evolutionary strategy gradient. Through goal-oriented exploration, robot learning skills were extended to cover different parameter environments.

Li et al. proposed a multi-modal incremental learning framework based on the teleoperation strategy to reduce the error between the reconstructed and expected trajectories, enabling the robot to accurately reproduce the demonstration task.

Duque-Domingo et al. proposed a novel method for deciding who the robot should pay attention to when interacting with multiple people during the process of interaction. The method is based on the receipt of different stimuli by a competitive network (see, say, pose, hoard talk, habituation, etc.), and then competing with each other to decide who to focus on (Duque-Domingo et al.).

The article by Navarro-Alarcon et al. presents a new scheme for approximating unknown sensorimotor robot models by using feedback signals only.

Cherubini et al. systematically reviewed the existing sensor-based control methods, and then discussed the problems, potential applications, and future research directions.

Ergonomics have a significant impact on productivity as well as the chronic health risks caused by inappropriate work postures and conditions. Peternel et al. proposed a new method for estimating and transferring ergonomic working states called a Binary Work-Condition Map to provide visual feedback on the working state of different arm structures. As well as combining the advantages of both the binary map and continuous map, these researchers proposed a Hybrid Work-Condition Map for ruling out unsuitable workspaces using the binary map approach, while rendering suitable workspaces by applying the continuous map approach (Peternel et al.).

All these methods help to improve the performance of human-robot interaction. The studies demonstrate the significant potential of combining machine-learning methods and sensor technology to visualize and interpret data, ultimately enhancing the ability of human and robot cooperation to complete related tasks.

## Author Contributions

BF wrote the manuscript. CF, LW, and PM helped to improve the manuscript. All authors contributed to the article and approved the submitted version.

## Conflict of Interest

The authors declare that the research was conducted in the absence of any commercial or financial relationships that could be construed as a potential conflict of interest.

